# Interaction of silicon and manganese in nutritional and physiological aspects of energy cane with high fiber content

**DOI:** 10.1186/s12870-022-03766-8

**Published:** 2022-07-29

**Authors:** Kamilla Silva Oliveira, Renato de Mello Prado, Mirela Vantini Checchio, Priscila Lupino Gratão

**Affiliations:** 1 Department of Agricultural Production Sciences, Sector of Soils and Fertilizers, Laboratory of Plant Nutrition, São Paulo State University (UNESP), Via de Acesso Prof. Paulo Donato Castellane, s/n, Jaboticabal, São Paulo, 14884-900 Brazil; 2 Department of Biology Applied to Agriculture, Laboratory of Plant Physiology, São Paulo State University (UNESP), Via de Acesso Prof. Paulo Donato Castellane, s/n, Jaboticabal, São Paulo 14884900 Brazil

**Keywords:** Abiotic stress, Oxidative stress, Hydrogen peroxide, Phenols, Superoxide dismutase, Guaiacol peroxidase, Uptake efficiency

## Abstract

**Background:**

Silicon (Si) is a multiple stress attenuator element in plants, however more research is needed to elucidate the actions in the plants defense system with low nutrition of manganese (Mn) for a prolonged period, and the attenuation mechanisms involved in the effects of Mn deficiency on energy cane with high fiber content. Thus, the objective of this study was to evaluate whether Si reduces the oxidative stress of the energy cane grown in low Mn in nutrient solution, to mitigate the effects of Mn deficiency, improving enzymatic and non-enzymatic defense, uptake of Mn the plant growth.

**Methods:**

An experiment was carried out with pre-sprouted seedlings *of Saccharum spontaneum* L. in a 2 × 2 factorial scheme in five replications in which the plants were grown under sufficiency (20.5 μmol L^−1^) and deficiency (0.1 μmol L^−1^) of Mn combined with the absence and presence of Si (2.0 mmol L^−1^) for 160 days from the application of the treatments. The following parameters were evaluated: accumulation of Mn and Si, H_2_O_2,_ MDA, activity of SOD and GPOX, total phenol content, pigments, and quantum efficiency of PSII.

**Results:**

Mn deficiency induced the oxidative stress for increase the H_2_O_2_ and MDA content in leaves of plants and reduce the activity of antioxidant enzymes and total phenols causing damage to quantum efficiency of photosystem II and pigment content. Si attenuated the effects of Mn deficiency even for a longer period of stress by reducing H_2_O_2_ (18%) and MDA (32%) content, and increased the Mn uptake efficiency (53%), SOD activity (23%), GPOX (76%), phenol contents, thus improving growth.

**Conclusions:**

The supply of Si promoted great nutritional and physiological improvements in energy cane with high fiber content in Mn deficiency. The results of this study propose the supply of Si via fertirrigation as a new sustainable strategy for energy cane cultivation in low Mn environments.

## Background

Manganese (Mn) is important in the photosynthetic process and antioxidant defense system of plants for its functions as enzyme activator, enzyme cofactor, and electron transporter [1]. However, soils with low moisture, aerated, and with high content of organic matter, as well as alkaline soils, have reduced Mn availability, inducing deficiency in crops [2] established in different soils worldwide [3]. Furthermore, Mn deficiency has also been reported in tropical regions due to anthropic activity, which resulted in an excessive amount of limestone on the soil surface [4, 5], aggravating the problem of deficiency in agricultural production.

Mn deficiency causes changes in oxidative metabolism, generating imbalance between the production and elimination of reactive oxygen species (ROS) by modulating the activities of enzymes that are responsible for the reduction of these compounds in plant cells, e.g., superoxide dismutase (SOD), which is a cofactor, and peroxidases [1, 5–8]. In addition to the enzyme system, other antioxidants, such as phenols [7, 9] and carotenoids [10] complement the non-enzyme antioxidant defense system. They need Mn to be synthesized, being reduced when there is deficiency of this micronutrient.

These alterations resulting from Mn deficiency trigger cell membrane oxidation by ROS, leading to lipid peroxidation [11]. Moreover, Mn deficiency in the plant metabolism disrupts the structure of thylakoids and chloroplasts and extends damage to photosynthetic pigments, reducing the contents of these molecules [12, 13]. It also impairs the functioning of Photosystem II (PSII), which is essential for the first phase of photosynthesis [14–16], ultimately stimulating ROS overproduction [17]. Thus, Mn deficiency is a serious nutritional disorder that compromises crop performance and limits growth.

There is little research on Mn in energy cane with high fiber content (*Saccharum spontaneum* L.). Due to this characteristic in its composition, the cultivation of this species has recently emerged when prioritizing the production of fibers in place of sucrose, presenting greater economic return than growing a species with high sucrose content, such as *Saccharum officinarum* L. [18], to increase energy cogeneration (biofuel and electricity), given the need to reduce CO_2_ emissions into the atmospheric air.

One way to minimize the effects of Mn deficiency on energy cane may be related to the use of silicon (Si). Si has been described as a beneficial element for plant species under biotic and abiotic stresses [19], especially for plants that have a high capacity to accumulate Si in their tissues, such as *Saccharum officinarum* L. and other Poaceae [20–22]. Although studies involving Si in the mitigation of abiotic stress have increased in recent years, they are still restricted involving the relationship of Si and Mn. This was found in a recent review, which indicated that the analysis of Mn and Si interactions aimed at mitigating the condition of deficiency are still scarce, being more elucidated when there is toxicity [23].

Thus, there are some indications that Mn deficiency could be mitigated by the induction of Si to activate the enzymatic antioxidant defense system in the plant by increasing SOD activity in sorghum plants, for example [8]. If Si decreases oxidative stress, it should impact the reduction of pigment degradation and increase the quantum efficiency of PSII, improving photosynthesis [24–26] and providing dry weight gains. What needs to be proven is whether the decrease in oxidative stress promoted by Si is sufficient to improve the physiological aspects of the plant, as this may vary according with the cultivation condition and the cultivated species.

Another effect of Si would be to increase the activity of enzymes responsible for Mn transport in cell membranes [27–30], favoring Mn accumulation in the plant shoot. However, this has not yet been studied in energy cane with high fiber content.

Clearly, there is little information on the possible actions of Si to mitigate Mn deficiency in plants cultivated in soils with low Mn availability, and this scenario can reduce the great potential for biomass production presented by energy cane to generate renewable energy, which is a global trend. Although there is evidence of the benefit of Si in mitigating Mn deficiency in an energy cane cultivar with low fiber content grown for only 58 days [31], further knowledge is needed. This occurs as the period of stress (Mn deficiency) that normally occurs in the energy cane cycle is longer, surpassing 150 days, and it is still unknown whether Si could mitigate this nutritional deficiency in this scenario.

Another important aspect is the emergence of new energy cane cultivars with a high content of lignocellulosic fiber (> 30%), as it is still unknown whether this fact could increase the benefit of Si in Mn-deficient plants. The importance of Mn in the synthesis of fiber components such as lignin is known [2], and Si has important effects on cell wall biosynthesis, lignification, and its mechanical function [32]. These facts can impact fiber formation and integrity, consequently impacting energy cane with high fiber content, but they have to be proven. Therefore, it is necessary to understand and unravel the mechanisms of Si that benefit the growth of energy cane with high fiber content cultivated in a Mn-deficient medium.

For this, it is pertinent to test the following hypotheses: (i) Mn deficiency, regardless of Si, causes important damage in physiological and nutritional aspects, indicating that energy cane with high fiber content is more sensitive to Mn deficiency; and (ii) Si attenuates the effects of Mn deficiency on energy cane by the sum of the benefits, that is, by increasing Mn uptake efficiency and reducing oxidative stress by acting on the enzymatic and non-enzymatic antioxidant defense system, consequently providing higher dry mass conversion and plant growth.

Therefore, this study aimed to evaluate whether Si can attenuate the effects of Mn deficiency in type II energy cane under low Mn conditions by reducing oxidative stress and acting in the enzymatic and non-enzymatic defense mechanisms, inducing improvements in photosynthetic functions and growth.

## Material and methods

### Installation of the experiment

The experiment with the type II energy cane genotype (*Saccharum spontaneum* L.) variety VX2 was developed in a greenhouse at São Paulo State University – UNESP, Jaboticabal campus, Brazil.

For installation of the experiments, pre-sprouted seedlings were prepared from mini-cuttings (5 cm long), and a bud was planted in a polypropylene tray filled with fine vermiculite. After the full emergence of the shoots (7 days after planting), Si (2.5 mmol L^−1^) was applied via fertigation at 4-day intervals for 50 days. On these occasions, the complete nutrient solution by [33] was also applied with Mn concentration modified to 13.65 μmol L^−1^. Also, the source was changed from Fe-EDTA to Fe-EDDHA. Then, the seedlings were cut at 30 cm height of the substrate for uniformity, and the application of the treatments was started. After 28 days of uniformity cut and start of treatments, the seedlings were transferred to polypropylene vessels that had a volume of 1.5 dm^3^ and had been filled with washed sand.

The treatments were composed of a 2 × 2 factorial scheme in 5 repetitions, arranged in random blocks. The treatments consisted of plants grown under Mn sufficiency (20.5 μmol L^−1^) and Mn deficiency (0.1 μmol L^−1^), combined with the absence and presence of Si (2.0 mmol L^−1^) supplied for 160 days. The source of Si was sodium and potassium silicate stabilized with sorbitol (SiNaKE) with 107.9 g L^−1^ of Si and 16.4 g L^−1^ of K_2_O. The nutrient solution by [33] was used with modification of Mn concentration. The pH value of the nutrient solution was adjusted to 5.5 ± 0.5 using an HCl or NaOH solution (1.0 mol L^−1^). The potassium concentration of the nutrient solution for treatments without Si was adjusted with potassium chloride. The meteorological data were measured inside the greenhouse during the growth period, and they corresponded to the average of the minimum and maximum values for relative humidity equal to 10% and 32%, and for air temperature values equal to 16ºC and 38ºC, respectively.

At the end of the experiment, at 153 days after the application of the treatments, biochemical and physiological evaluations were performed in the plants.

The plant is owned by author (Kamilla Silva Oliveira). The permission is already provided.

### Analyses

#### Mn and Si accumulation, Mn uptake efficiency

Mn content of the shoots and roots was determined according to the method proposed by [34]. Si content was determined on the basis of the digestion of the samples, following the method described by [35], and concentration was determined using the reading performed by a spectrophotometer at 410 nm, as described by [36]. Total accumulation of Mn and Si was determined by the sum of the dry weight product and the content of the elements in the shoots and roots. At the end, Mn uptake efficiency was determined using the ratio of total Mn accumulation in the plant and dry weight of roots [37].

#### Determination of H_2_O_2_ content

Hydrogen peroxide (H_2_O_2_) content was estimated in leaf collected at 159 days after of the treatments, according to the methods of [38]. Absorbance was read at 390 nm, and H_2_O_2_ content was determined on the basis on a standard curve of H_2_O_2_ concentration expressed in μMol g^−1^ of fresh weight.

#### Lipid peroxidation (MDA)

To determine lipid peroxidation, the first fully expanded leaf was collected at 159 days after application of the treatments, following the methods described by [39]. Malondialdehyde concentration (MDA) was determined by reading on a spectrophotometer between 535 and 600 nm, and the data were calculated based on the extinction coefficient of 1.55 × 10^−5^ mol^−1^ cm^−1^, with MDA results expressed in nMol g^−1^ fresh weight [40].

#### Protein extraction

Total soluble protein was extracted using 1.0 g of the first fully developed leaf collected at 159 days after the application of the treatments and homogenized in a cooled mortar and pestle with 100 mM potassium phosphate buffer (pH 7.5), 1 mM ethylenediaminetetraacetic acid (EDTA), 3 mM DL-dithiothreitol, and 5% (w/v) insoluble polyvinylpolypyrrolidone in a 3:1 volume/fresh weight ratio [41]. The material was centrifuged at 10,000 × g for 30 min, and the supernatant was stored at − 80 °C for later determination of the activities of the SOD and GPOX enzymes. Protein concentration was determined according to the [42] method with bovine serum albumin as a standard, expressed in mg mL^−1^ protein.

#### Superoxide dismutase (SOD) assay

SOD activity (SOD, EC 1.15.1.1) was determined in a spectrophotometer, as described by [43], and the reaction was conducted in a reaction chamber (box) under the lighting of 15 W fluorescent lamp at 25 °C. The reading was performed at 560 nm and SOD activity was expressed in U mg^−1^ protein.

#### Guaiacol peroxidase (GPOX) assay

GPOX activity (GPOX, EC 1.11.1.7) was determined in a mixture with phosphate citrate buffer at pH 5.0 (0.2 dibasic sodium phosphate: 0.1 citric acid), 0.5% guaiacol, and the extract. The activity was evaluated by monitoring absorbance at a wavelength of 450 nm [44].

#### Determination of total phenol content

Total phenolic compounds were extracted using 0.1 g of fresh fully expanded leaves collected at 154 after the application of the treatments. After extraction, absorbance was read in a spectrophotometer at wavelength of 765 nm, and the content was determined based on the standard curve with gallic acid, expressed in g gallic acid equivalent (GAE) 100 g^−1^ [45].

#### Chlorophyll a, Chlorophyll b and carotenoid contents

Pigment content was determined using leaf discs taken from the middle third of the leaf blade, completely expanded at 154 after the application of the treatments, according to the methodology proposed by [46]. Readings at 663 nm for chlorophyll a (Chl *a*), 647 nm for chlorophyll b (Chl *b*), and 470 nm for carotenoids, were performed on a Beckman DU 640 spectrophotometer, with contents defined based on fresh weight.

#### Quantum efficiency of photosystem II (Fv/Fm)

The quantum efficiency (Fv/Fm) of PSII was obtained by measuring chlorophyll *a* fluorescence using a fluorimeter, model Opti-sciences—Os30P. The measure was performed at 153 days after the application of the treatments on the middle third of the leaf + 1 (first complete leaf with visible sheath) between 7:30 and 8:30 a.m. To this end, the sampled region was submitted to the dark for adaptation at least 30 min before excitation of the red-light pulse of 1 s.

#### Growth

At 160 days after the application of treatments, the leaf area was measured with a leaf area-measuring device (LI-3100, Li-Cor, USA). The result was expressed in cm^2^. To obtain the root and total plant dry mass the plants were cut at the substrate level and separated the shoots and roots to compose roots dry mass and total dry mass. The fractions were washed with detergent solution (0.1% v/v), acid solution with HCl 1.0 mol L^−1^ (0.3% v/v) and deionized water. The samples were placed in paper bags and dried in a forced-air circulation oven (65 ± 2 ºC) to constant weight. Dry weight of the whole plant was determined in grams.

### Statistical analysis

The normality test (Shapiro – Wilk test) and homogeneity of variances test (Levene’s test) were performed on the data using software libre R 4.0.3. The significant differences between treatment were assessed using an ANOVA and Tukey–test (*p* < 0.05 significance level) in the SAS statistical program (Cary, NC, USA). The interactions were sliced even when not significant and the elaborated graphs in SigmaPlot 14.0 (Systat Software, Inc, San Jose, CA).

## Results

### Si potentiate the Mn nutrition increasing Mn total content and Mn uptake efficiency in energy cane deficient and sufficient

**Fig. 1:**
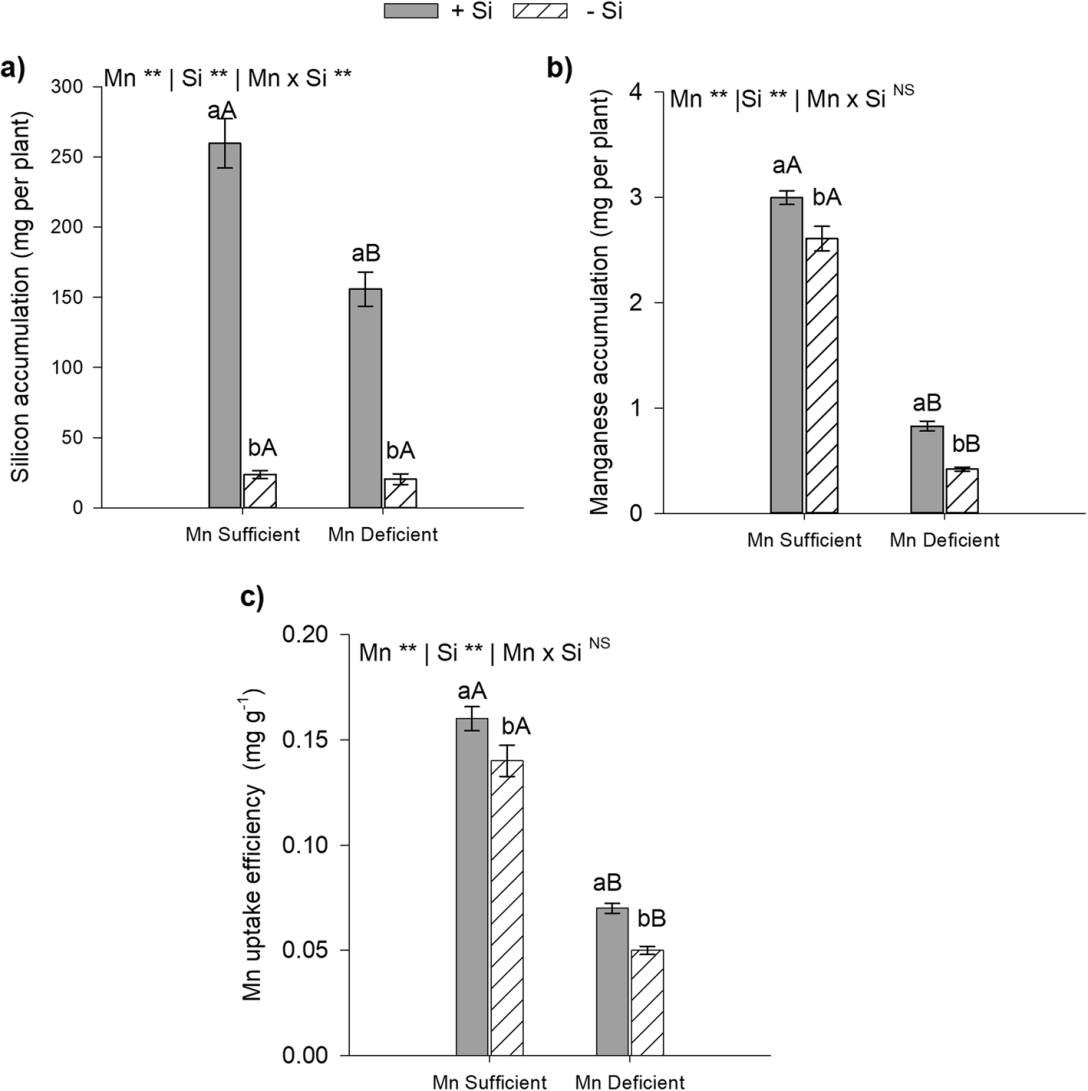
Accumulation of Si (**a**) and Mn (**b**), Mn uptake efficiency (**c**) of type II energy cane (*Saccharum spontaneum* L.), cultivated in sufficient and deficient nutrient solution of Mn in the absence (-Si) and in the presence (+ Si) of Si. **Significant at 1% probability; *significant at 5% probability, and.^ns^ non-significant by the F-test. Different lowercase letters compare Si conditions under the same Mn condition, while different uppercase letters compare Mn conditions under the same Si condition (*p* < 0.05 by Tukey’s test). Bars represent the standard error of the mean, *n* = 5

In this study the Si provided via nutrient solution increased the accumulation of this element in Mn-sufficient and Mn-deficient energy cane plants in comparison to those that did not receive the element (*p* < 0.01). However, the results showed that in Mn-deficient plants, in comparison to Mn-sufficient ones, in the absence or presence of Si, there was a decrease in Si accumulation in the plant (*p* < 0.01; Fig. 1a). This suggests that inadequate Mn nutrition influences Si absorption and accumulation, fact confirmed by the significant effect of the Mn x Si interaction. Si increased Mn accumulation independent of the nutritional status of Mn of the plants (*p* < 0.01). The Si provided increased Mn accumulation by 97 and 15% in plants grown under deficiency and sufficiency of this micronutrient, respectively (Fig. 1b).

Mn uptake efficiency by plants in the presence or absence of Si decreased in Mn-deficient plants in comparison to Mn-sufficient ones (*p* < 0.01). However, the supply of Si in the nutrient solution increased plant uptake efficiency by 18% and 53% in plants grown under micronutrient sufficiency and deficiency, respectively (Fig. 1c). This indicates a positive relationship of Si in nutrition with Mn.

### Si alleviate Mn deficiency by reducing oxidative stress and increasing enzymatic and non-enzymatic antioxidant defense in energy cane plants

**Fig. 2:**
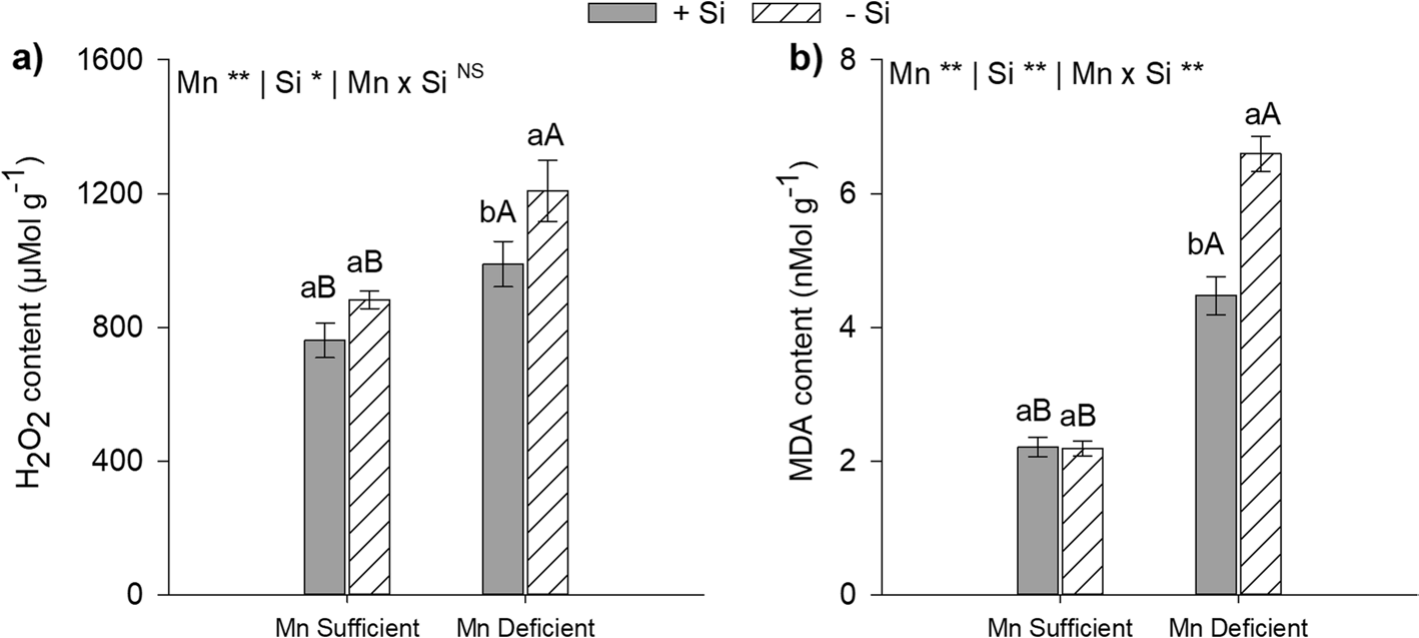
Hydrogen peroxide (**a**) and malondialdehyde (**b**) content in leaves of type II energy cane plants (*Saccharum spontaneum* L.), cultivated in Mn-sufficient and Mn-deficient nutrient solution in the absence (-Si) and in the presence (+ Si) of Si. **Significant at 1% probability; *significant at 5% probability, and ^ns^ non-significant by the F-test. Different lowercase letters compare Si conditions under the same Mn condition, while different uppercase letters compare Mn conditions under the same Si condition (*p* < 0.05 by Tukey’s test). Bars represent the standard error of the mean, *n* = 5

Mn deficiency in comparison to Mn sufficiency in the absence or presence of Si increased the contents of H_2_O_2_ and MDA in energy cane plants indicating oxidative stress and cellular damage (*p* < 0.01). By contrast, significant reductions in ROS content (H_2_O_2_) and indicator of lipid peroxidation (MDA) occurred in leaves when Si was added to the cultivation solution. The presence of Si in the cultivation solution in Mn-deficient plants decreased the content of H_2_O_2_ and MDA by 18% and 32%, respectively (Fig. 2a and 2b).

**Fig. 3:**
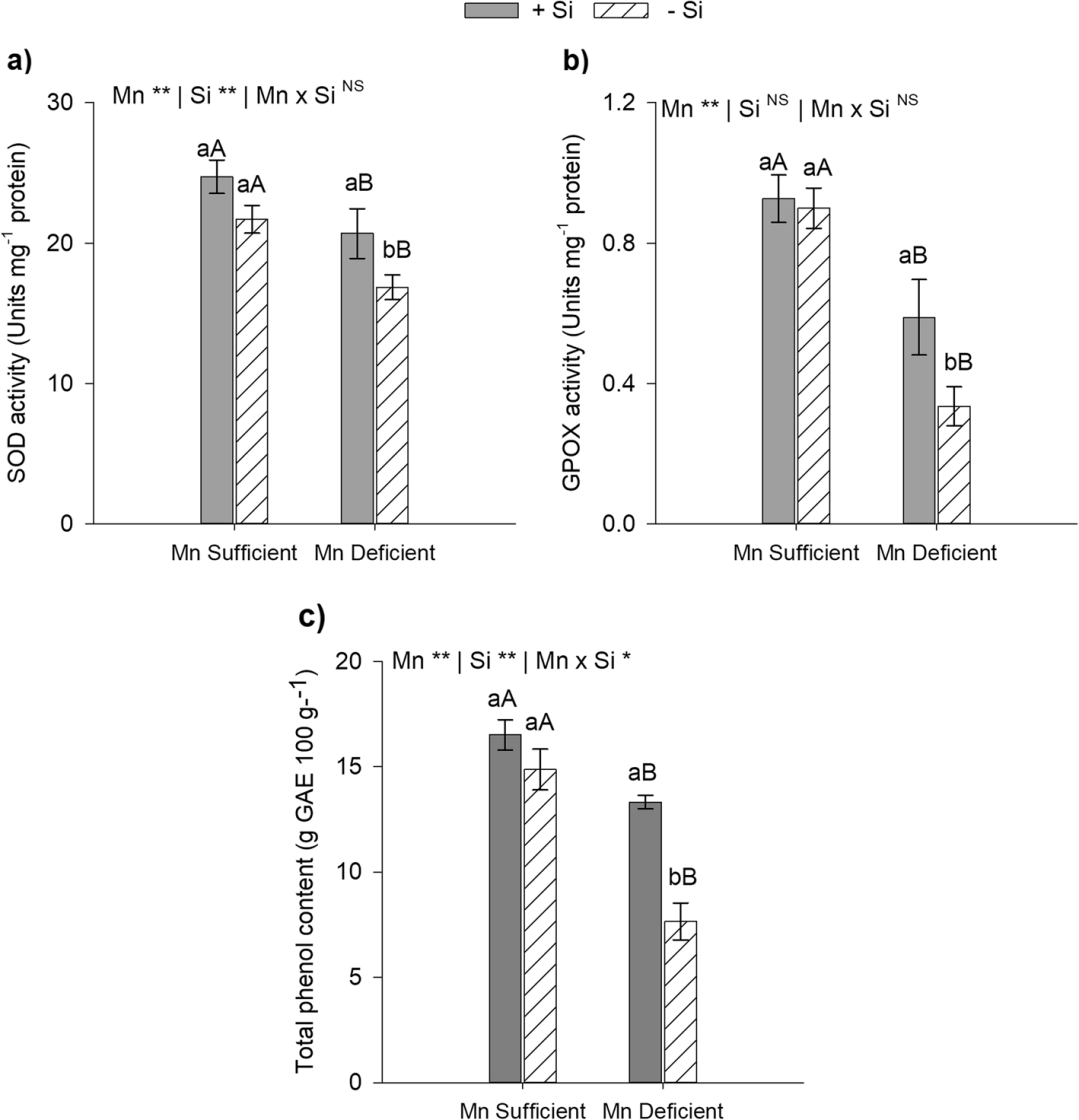
Activity of superoxide dismutase (SOD) (**a**), guaiacol peroxidase (GPOX) (**b**) and total phenols content (**c**) in leaves of type II energy cane plants (*Saccharum spontaneum* L.), cultivated in Mn-sufficient and Mn-deficient nutrient solution in the absence (-Si) and in the presence (+ Si) of Si. **Significant at 1% probability; *significant at 5% probability, and ^ns^ non-significant by the F-test. Different lowercase letters compare Si conditions under the same Mn condition, while different uppercase letters compare Mn conditions under the same Si condition (*p* < 0.05 by Tukey’s test). Bars represent the standard error of the mean, *n* = 5

To investigate the effects of Si on the defense system of plants subjected to Mn deficiency stress, we evaluated the activity of SOD and GPOX enzymes and the total phenols content (Fig. 3). The activity of SOD and GPOX enzymes was decreased in Mn-deficient plants in comparison to Mn-sufficient plants in the presence and absence of Si (*p* < 0.01). However, the antioxidant enzymes activity in the presence of Si were upregulated compared to the absence of Si in Mn-deficient energy cane plants. The presence of Si in the nutrient solution increased the activity of SOD and GPOX by 23% and 76%, respectively (Fig. 3a and b).

Total phenolic content of leaves decreased under Mn deficiency in comparison to Mn-sufficient plants in the presence or absence of Si. However, the application of Si in comparison to its absence increased the total content phenolic of plants under Mn deficiency by 74% (*p* < 0.01; Fig. 3c).

### Si in energy cane Mn-deficient plants improves pigment content, photochemical efficiency and increases growth

**Fig. 4:**
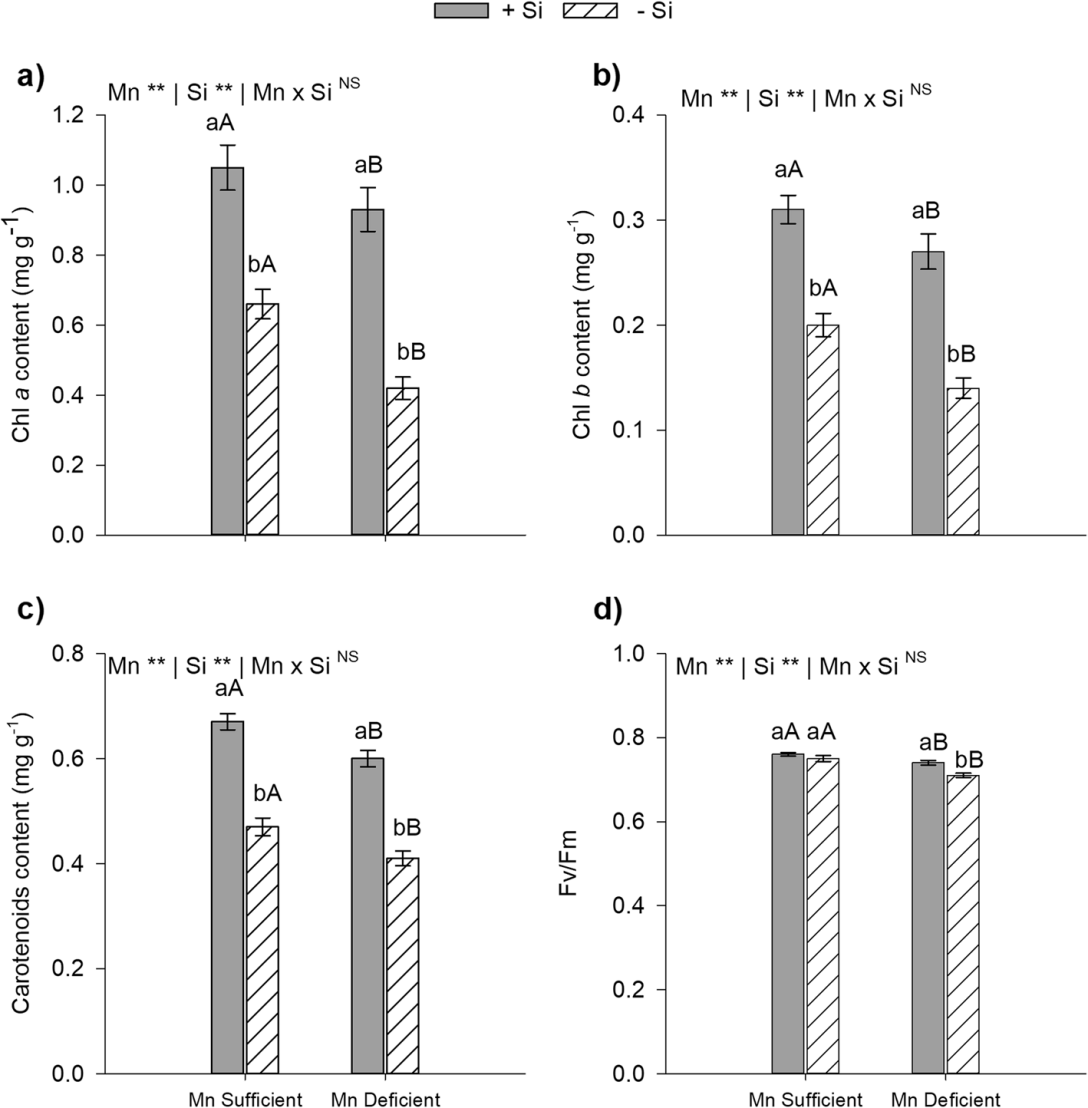
Content of chlorophyll *a* (**a**), chlorophyll b (**b**), carotenoids (**c**) and, quantum efficiency of photosystem II (**d**) of type II energy cane plants (*Saccharum spontaneum* L.), cultivated in Mn-sufficient and Mn-deficient nutrient solution in the absence (-Si) and in the presence (+ Si) of Si. **Significant at 1% probability; *significant at 5% probability, and ^ns^ non-significant by the F-test. Different lowercase letters compare Si conditions under the same Mn condition, while different uppercase letters compare Mn conditions under the same Si condition (*p* < 0.05 by Tukey’s test). Bars represent the standard error of the mean, *n* = 5

Plants grown in a nutrient solution under Mn deficiency in the absence or presence of Si presented lower pigment contents (chlorophylls *a, b* and carotenoids) and lower quantum efficiency of photosystem II (*p* < 0.01). By contrast, our results indicated that in Mn-sufficient and Mn-deficient plants, the presence of Si in the nutrient solution increased the content of Chl *a* (58% and 120%)*,* Chl *b* (58% and 96%) and carotenoids (44% and 47%), respectively (Fig. 4a, b and c) (*p* < 0.01). Si applied in the nutrient solution increased the quantum efficiency of PS II, but only in Mn-deficient plants (Fig. 4d) inducing additional physiological benefit to plants beyond antioxidant defense.

**Fig. 5:**
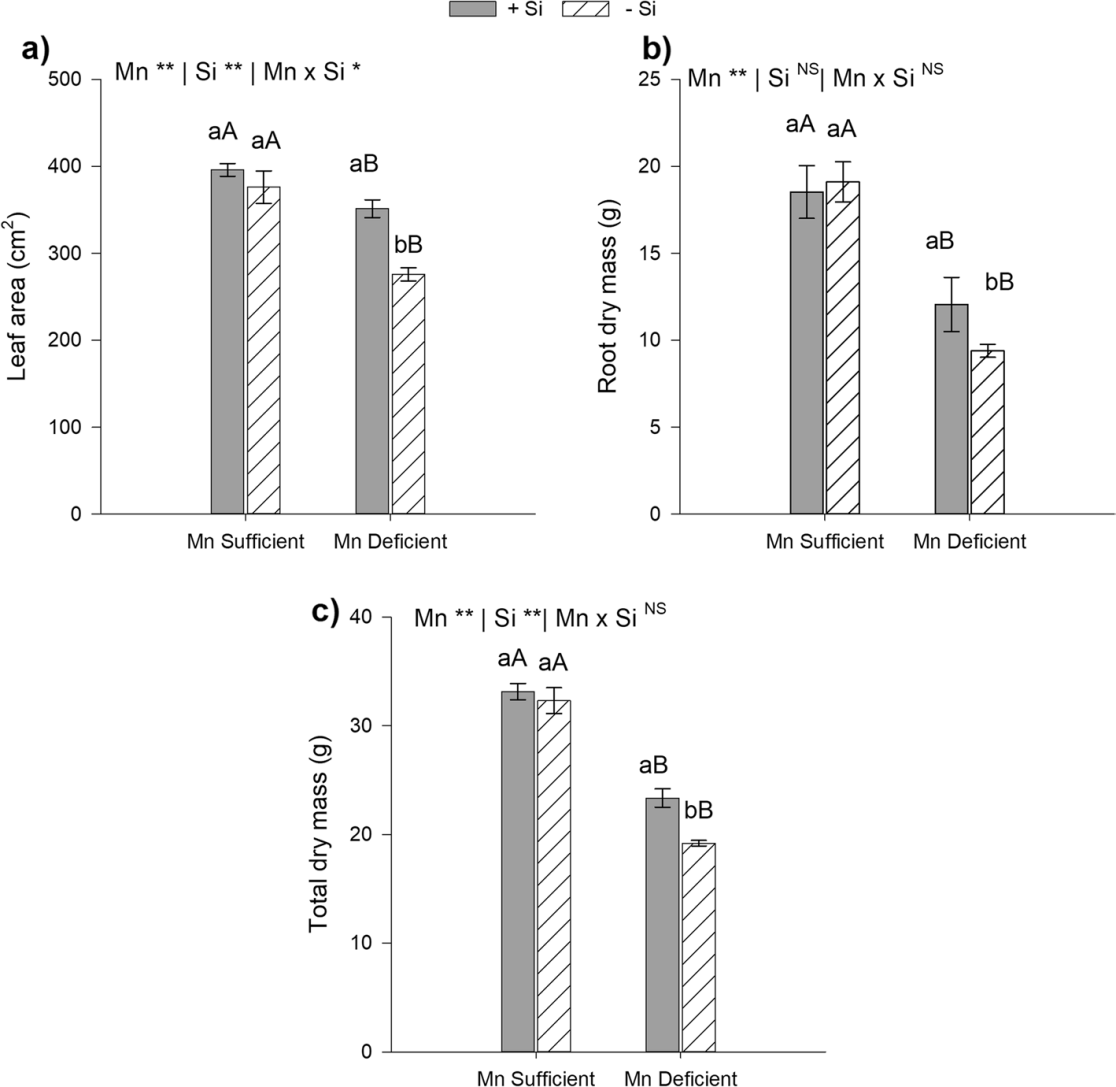
Leaf area (**a**)**,** root dry mass (**b**) and total dry mass (**c**) of type II energy cane plants (*Saccharum spontaneum* L.), cultivated in Mn-sufficient and Mn-deficient nutrient solution in the absence (-Si) and presence (+ Si) of Si. **Significant at 1% probability; *significant at 5% probability, and ^ns^ non-significant by the F-test. Different lowercase letters compare Si conditions under the same Mn condition, while different uppercase letters compare Mn conditions under the same Si condition (*p* < 0.05 by Tukey’s test). Bars represent the standard error of the mean, *n* = 5

The results indicated that Mn deficiency negatively affected plant growth (*p* < 0,01). Mn-deficient plants, in comparison to Mn-sufficient ones, in the presence or absence of Si, had a decrease in leaf air, root dry mass and dry weight (Fig. 5). In this study the supply of Si in Mn-deficient plants increased leaf area by 27% in Mn-deficient plants (Fig. 5a), 29% in root dry mass (Fig. 5b) and dry weight by 22% (Fig. 5c). This indicates an ameliorative effect of Si on plant growth under Mn deficiency stress.

## Discussion

The efficiency of nutrient uptake is partly modulated by root characteristics and by the amount of the specific nutrient applied [47]. It was found that energy cane plants under Mn deficiency presented low Mn accumulation and uptake efficiency, which consequently led to biological damage to these plants.

This nutritional disorder of Mn promotes an increase in ROS, e.g., H_2_O_2,_ which was observed in sorghum plants [8], maize [7], and also energy cane. Therefore, plants under Mn deficiency are susceptible to oxidative stress caused by ROS overproduction due to the poor ability of plants to induce antioxidant defense mechanisms. In the present study, an important impairment was found in the enzyme and non-enzyme antioxidant system of Mn-deficient plants.

The decrease in SOD activity occurred in plants with nutritional deficiency as Mn is a constituent of this enzyme [1, 13], which was also reported by [8] in sorghum plants and by [31] in sugarcane. Moreover, there was a decrease in glutathione peroxidase (GPOX) activity, which is important for H_2_O_2_ dismutation in water and oxygen [48]. The decrease in the activity of another peroxidase (APX) was the main response of the plant to Mn deficiency [6], thus consisting of an important characteristic of response to this nutritional disorder. GPOX belongs to the class of enzymes that catalyze H_2_O_2_ reduction by removing electrons from donor molecules such as phenolic compounds [49], whose synthesis requires Mn [50] and which may play a relevant role in the reduction of enzyme activity.

Our data showed that Mn deficiency in energy cane decreased the total phenolic content. This may have occurred as Mn activates specific enzymes involved in phenol biosynthesis [9, 50]. Mn stabilizes the active conformation of the enzyme phenylalanine ammonia lyse (PAL), which catalyzes the non-oxidative deamination of phenylalanine in cinnamic acid [51], promoting the formation of phenols and other compounds. This loss of Mn deficiency in this non-enzymatic antioxidant compound also contributed to increase the H_2_O_2_ content, as phenols can reduce lipid peroxidation by eliminating active molecular oxygen and H_2_O_2_ in the plant metabolism [48].

The lower activity of GPOX associated with a lower phenol content, as found in energy cane, may have led to H_2_O_2_ accumulation_._ In addition, a decrease in SOD activity due to the lack of Mn can induce the accumulation of another harmful ROS, namely: O_2_^•−^ [13, 48, 52]. The accumulation of H_2_O_2_ and O_2_^•−^ in Mn-deficient plants was recently demonstrated in wheat [53]. The accumulation of reactive species results in lipid peroxidation, as evidenced by the increase in MDA content in energy cane plants. This is the result of the lipid degradation of cell membranes and an evidence of oxidative damage [54] that has been reported as an effect of Mn deficiency in other grasses [8, 11].

Oxidative stress decreased leaf pigments of Mn-deficient energy cane plants due to membrane degradation of thylakoids and chloroplasts [55] and a decrease in the number of chloroplasts [56]. In addition, Mn is required for the synthesis of lipids and proteins, which are structural components of this organelle [9, 13], as well as of carotenoids [57]. In Mn deficient-plants, plastid and chloroplast gene transcription are reduced, which reduce the content of integral proteins in the organelle and their synthesis, affecting the integrity of the chloroplast and disorganizing the thylakoid membrane system [12].

The effects of Mn deficiency on pigment concentration are also a consequence of the lower quantum efficiency of PSII [58], a fact also found in energy cane. This results from the function of Mn as a component of the catalytic Mn_4_Ca cluster of the photochemical stage of photosynthesis responsible for water photooxidation [13]. When Mn is deficient, it destabilizes and disintegrates the Mn complex in PSII [14]. In addition, it increases the formation of singlet oxygen (^1^O_2_), which can degrade chlorophylls and the D1 protein of PSII [59] if not eliminated by carotenoids and tocopherols in the chloroplasts [60].

This damage caused by Mn deficiency, impairing the energy cane plant metabolism, explains the decrease of growth variables. It is noteworthy that Mn deficiency has a strong impact by reducing the growth of plant roots and shoots, which was observed in barley plants [61]. Similarly, there were important reductions in the leaf area and dry mass of roots and shoots of energy cane, a fact that has also been described for other species [8, 14, 62].

Our study still evidences that energy cane with high fiber content is much more sensitive to Mn deficiency. This was evidenced as the deficiency of Mn in relation to plants under sufficient nutrition of Mn induced a decrease in plant mass production (total) of energy cane with high fiber content equal to 62%, which was much higher than that observed for energy cane with low fiber content, which reached only 22%, according to [31]. This occurs as Mn is an important nutrient for photosynthesis [63] and for the synthesis pathway of vegetable fiber, especially lignin [50, 64]. Therefore, it is a nutrient that is very limiting to the production of this species when deficient, showing the importance of its adequate management. In this case, there is a need for strategies to mitigate this nutritional stress, among which the most promising is the use of Si.

We found that energy cane presents high Si absorption capacity regardless of the nutritional status in terms of Mn in the plant. Thus, the Si absorption capacity in energy cane indicates that it bears resemblance to sugarcane (*S. officinarum* L.), which is a Si-accumulating plant [20, 65]. This occurs as Poales present specific transporters in the cell membranes of the roots that are efficient for the absorption of Si [21]. More recently, it was found that that the presence of transporters such as intrinsic proteins similar to nodulin 26 (NIP-III) in some species of Poales is a result of species evolution and indicates a high capacity to accumulate Si [22].

The high ability of energy cane to accumulate Si in the shoot increases the possibility of the biological benefits of the element in this species. The first benefit is the contribution of Si in increasing the accumulation and uptake efficiency of Mn regardless of the plant nutritional status. The increase in Mn absorption induced by Si was reported only in sugarcane under adequate Mn conditions and in energy cane with lower fiber content (type I) under Mn sufficiency and deficiency [31]. Thus, even this species with higher fiber content, such as the one analyzed in our study, presents benefits from Si in nutrition with Mn in both conditions of Mn supply. Previously, similar results were reported in sorghum plants grown under Mn deficiency [15] and in corn and wheat plants grown without deficiency of this micronutrient [66]. As the Mn uptake efficiency in this study takes into consideration the potential to accumulate Mn in the plant in relation to the amount of roots [37], it is clear that the benefit of Si in providing a higher Mn uptake efficiency was a reflection of the increment in the accumulation of Mn, especially in plants in the condition of deficiency (97%).

The increase in Mn absorption promoted by Si may have occurred due to the effect of the beneficial element on the modulation of H + -ATPase activity by the expression of genes that encode these proteins, which are involved in generating an electrochemical gradient for the active absorption of nutrients [27, 67, 68]. In addition, Si increases the activity of Mn-specific active transporters [2, 30, 69]. This effect of Si draws attention, as NRAMP proteins (Nramp 6 and Nramp 1) were recently identified as responsible for playing critical roles in the absorption and utilization of Mn in plants subjected to conditions of low availability of the micronutrient [17]. Therefore, the fact that Si had a positive effect on this same family of transporters in other studies [30] reflects an important benefit of Si against the potential nutritional disorder of Mn deficiency.

In the face of limited availability, plants need to be efficient in acquiring Mn through the roots to increase stress tolerance [2]. Thus, the effect of Si in enhancing this mechanism reflects an additional benefit to energy cane plants subjected Mn deficiency as a form of adaptation, also improving their performance in soils with adequate Mn fertilization.

It is also possible that the Mn that was absorbed in the first stage of the experiment for fifty days was remobilized to the plant shoot—a fact that was reported for iron in cucumber, barley, and sorghum plants [29, 70, 71]. These authors indicated that there was an increase in the expression of Si-induced genes encoding the biosynthesis of nicotianamine and in the YSL transporters responsible for the discharge of Fe into the phloematic vessels that reach the plant shoots. This can occur when Mn is precipitated in the apoplast or connected to the cell wall, being subsequently remobilized when there is a deficiency imposed to the plants [72]. In barley plants, the use of Si increased the Fe content in young leaves of deficient plants, which was associated with the increased expression of genes involved in Fe acquisition [29]. In addition, an increase in the translocation efficiency and accumulation of Fe in young leaves by Si was also observed in Fe-deficient sorghum plants [71].

We showed that the contribution of Si to energy cane plants is provided by the increase of Mn content in the shoot of plants deficient in this micronutrient, which was associated with the activation of the antioxidant enzymes of the defense system due an increase in the activity of SOD, GPOX and non-enzymatic antioxidants (phenols and carotenoids). This occurs as Mn is one of the cofactors of the SOD enzyme, composing the Mn-SOD isoform of this enzyme [1] and participating in the synthesis of phenols [9] and carotenoids by the activation of the phytoene synthetase enzyme in the synthesis of isoprenoids, which are precursors of these pigments [57].

The activity of SOD potentiated by Si possibly reduced the accumulation of the superoxide radical O2^• −^ and promoted the formation of H_2_O_2_ through the catalysis of this enzyme. Furthermore, it was observed that GPOX activity potentially increased (76%) with the supply of Si, which is possibly associated with increased SOD activity to reduce the H_2_O_2_ content, maintaining homeostasis between production and promoting the elimination of ROS. In an extensive review by [73] on the effects of Si on antioxidant systems under various abiotic stresses, the authors concluded that the main actions of Si were associated with the increased activity of enzymes involved in the transformation of H_2_O_2_ into water (APX and CAT). In our study, the effect of Si on the elimination of this ROS was made clear by the increase in GPOX activity and phenol content (74%), both occurring with the function of eliminating H_2_O_2_ [48] due to another abiotic stress – the nutritional deficiency.

The effect of Si on the increase of enzyme antioxidant activity was found in sorghum plants under Mn deficiency [8]. Si did not effectively affect the activities of SOD and GPOX enzymes in energy cane with low fiber content, but it benefited the non-enzymatic response by increasing the phenol content [31], which was also observed in plants under nutritional stress of other species [74, 75]. This may be associated, in addition to the longer exposure time to the deficiency, to the sensitivity of the species. Thus, in the face of damage, the response of all antioxidant components evaluated was potentiated by Si, including enzymes, which suggests that Si can benefit the metabolism of plants under Mn deficiency even in longer periods and in plants under severe deficiency. A similar fact was observed in barley plants sensitive to Al-induced stress and treated with Si, resulting in higher production of antioxidant components than in more tolerant plants [74].

These antioxidant mechanisms potentiated by Si in Mn-deficient energy cane plants decreased the overproduction and accumulation of H_2_O_2_ contents and possibly of other ROS, reducing the MDA content. In this scenario, Si decreased membrane degradation in photosynthesizing pigments [24, 29, 71, 76], which occurred in energy cane as there was an increase in the content of Chl *a*, Chl *b*, and carotenoids in Mn-deficient plants.

In Mn-sufficient plants, the application of Si increased leaf pigments, as the micronutrient is involved in the synthesis of chlorophyll and proteins, as well as in chloroplast integrity [9, 13]. However, the use of Si in these plants did not affect lipid peroxidation and quantum efficiency of PSII, indicating that these plants did not present oxidative stress. On the other hand, in Mn-deficient plants, the benefit of Si was evidenced by increasing the quantum efficiency of PSII of energy cane plants, which was also reported in sorghum and corn plants by [15] and recently in soybeans plants [16]. The fact that there was an improvement in electron transport in Mn-deficient plants through Si may have reduced ROS accumulation, since both the preservation and improvement in electron transport in PSII maintained the formation of singlet oxygen, superoxide ions, H_2_O_2_, and hydroxyl radicals [77].

The sum of these results resulted in a greater growth of plants under Mn deficiency, indicating an attenuation of the effects of Mn-deficiency by Si. The root, as the main element of nutrient absorption, was benefited in the presence of Si in Mn-deficient plants. This was a result of increased Mn absorption, as the nutrient influences root growth by participating in lipid metabolism, gibberellic acid production, and carbohydrate flux [63]. Similarly, the leaf area and the total dry mass of the plant were benefited by the nutritional, biochemical, and physiological improvements, increasing the conversion to dry mass.

The first hypothesis of this study, which reported that Mn deficiency, regardless of Si, causes important damage in physiological and nutritional aspects, indicating that energy cane is sensitive to Mn deficiency, can be accepted. Thus, in a Mn-deficient plant, Si is not capable of fully reversing the nutritional and physiological damage, promoting growth that is similar to that of a Mn-sufficient plant. This is easy to understand as Si does not replace the nutritional functions of Mn, but attenuates the effects of this nutritional disorder in the plant. However, the biological effects of Si are clearly evidenced in Mn-deficient plants, as it is known that this element benefits plants under stress conditions [19], proving the second hypothesis of this study.

The present study proves the benefits of Si in Mn-deficient plants, as it increases the absorption of the micronutrient associated with the modulation of antioxidant defense systems, decreases the production of H_2_O_2_ and lipid peroxidation, and increases the contents of photosynthesizing pigments, avoiding oxidative damage and increases the leaf area and dry weight of energy cane plants with high fiber content.

The results of Si in attenuating Mn deficiency in Poaceae, which are Mn-demanding plants, are promising. In addition, plants without Mn deficiency can also benefit from Si, which opens perspectives for further research to check if these benefits can reach non-Poaceae plants with or without nutritional deficiency.

## Conclusion

Silicon attenuates the effects of manganese deficiency in energy cane with high fiber by increasing Mn uptake efficiency, reducing oxidative stress by increasing enzyme and non-enzyme antioxidant compounds, improving quantum efficiency in photosynthesis, photosynthesizing pigments and growth. The results of this study propose the supply of Si via fertirrigation as a new sustainable strategy for energy cane cultivation in low Mn environments.

## Abbreviations

Mn: Manganese
Si: Silicon
ROS: Reactive oxygen species
SOD: Superoxide dismutase
GPOX: Guaiacol peroxidase
MDA: Malondialdehyde
H_2_O_2_: Hydrogen peroxide
PSII: Photosystem II
Chl a: Chlorophyll a
Chl b: Chlorophyll b
PAL: Phenylalanine ammonia lyse
^1^O_2_: Singlet oxygen
O2^−^: Superoxide radical
